# Estrés oxidativo en células endoteliales inducido por el suero de mujeres con diferentes manifestaciones clínicas del síndrome antifosfolípido

**DOI:** 10.7705/biomedica.4701

**Published:** 2019-12-30

**Authors:** Manuela Velásquez, Manuel Alejandro Granada, Julián Camilo Galvis, Ángela María Álvarez, Ángela P. Cadavid

**Affiliations:** 1 Grupo de Reproducción, Departamento de Microbiología y Parasitología, Facultad de Medicina, Universidad de Antioquia, Medellín, Colombia Universidad de Antioquia Departamento de Microbiología y Parasitología Facultad de Medicina Universidad de Antioquia Medellín Colombia; 2 Grupo de Estudio Trombosis, Facultad de Medicina, Universidad de Antioquia, Medellín, Colombia Universidad de Antioquia Grupo de Estudio Trombosis Facultad de Medicina Universidad de Antioquia Medellín Colombia

**Keywords:** síndrome antifosfolípido, estrés oxidativo, morbilidad, embarazo, trombosis, antioxidantes, Antiphospholipid syndrome, oxidative stress, morbidity, pregnancy, thrombosis, antioxidants

## Abstract

**Introducción.:**

El síndrome antifosfolípido se caracteriza por la presencia persistente de anticuerpos antifosfolípidos y manifestaciones clínicas de trombosis o morbilidad gestacional, las cuales se asocian con estrés oxidativo y disfunción endotelial.

**Objetivo.:**

Evaluar los marcadores de estrés oxidativo en células endoteliales, inducidos por el suero de mujeres con diferentes manifestaciones clínicas del síndrome antifosfolípido y analizar la capacidad antioxidante de los sueros.

**Materiales y métodos.:**

Se incluyeron 48 mujeres que fueron clasificadas así: presencia de anticuerpos antifosfolípidos y criterios clínicos de morbilidad gestacional, trombosis vascular o ambas. Como grupos control se incluyeron mujeres negativas para anticuerpos antifosfolípidos. En un modelo in vitro de células endoteliales estimuladas con los sueros de las mujeres del estudio, se determinaron algunos marcadores de estrés oxidativo por citometría de flujo. También, se analizó la capacidad antioxidante de los sueros incluidos.

**Resultados.:**

Los sueros de los grupos de mujeres con síndrome antifosfolípido que presentaban trombosis, con morbilidad gestacional o sin ella, generaron un incremento significativo (p<0,05 y p<0,001) en los marcadores de estrés oxidativo endotelial, en contraste con el control de suero humano normal. No se observaron diferencias en el efecto de los sueros de los diferentes grupos de estudio sobre la lipoperoxidación endotelial. Tampoco se encontró diferencia en la actividad antioxidante de los sueros.

**Conclusión.:**

El estrés oxidativo mitocondrial en el endotelio se asocia con la presencia de trombosis. Sin embargo, cuando esta se asocia con morbilidad gestacional, también se genera estrés oxidativo intracelular.

## Abstract (en)

**Introduction:**

The antiphospholipid syndrome is characterized by the persistent presence of antiphospholipid antibodies and clinical manifestations of thrombosis or gestational morbidity that are associated with oxidative stress and endothelial dysfunction.

**Objective:**

To evaluate markers of oxidative stress in endothelial cells induced by the serum from women with different clinical manifestations of the antiphospholipid syndrome, and to analyze the antioxidant capacity of the sera.

**Materials and methods:**

We included 48 women who were classified as follows: presence of antiphospholipid antibodies and clinical criteria of gestational morbidity alone, vascular thrombosis only, and gestational morbidity/vascular thrombosis. Control groups included antiphospholipid antibodies negative women. In an in vitro model of endothelial cells stimulated with sera from women included in the groups, some markers of oxidative stress were determined by flow cytometry. The antioxidant capacity in the sera of these women was analyzed.

**Results:**

The sera from the groups of women with antiphospholipid syndrome that presented thrombosis, with or without gestational morbidity, generated a significant increase (p<0,05 and p<0,001) in endothelial oxidative stress markers in contrast to the control of normal human serum. There were no differences in the effect of the sera from the different study groups on endothelial lipid peroxidation. Also, there was also no difference in the antioxidant activity of the sera.

**Conclusion:**

Mitochondrial oxidative stress in the endothelium is associated with the presence of thrombosis; instead, its association with gestational morbidity generates intracellular oxidative stress.

El síndrome antifosfolípido es una enfermedad autoinmunitaria que se caracteriza por manifestaciones clínicas de trombosis o morbilidad gestacional y la presencia de anticuerpos antifosfolípidos ^1^. Estos se consideran responsables del 10 al 25 % de los casos de aborto recurrente y, además, su prevalencia en mujeres con diagnóstico de morbilidad gestacional es del 5 al 50 % ^2^. Según un estudio reciente en el cual se hizo seguimiento a 1.000 pacientes con síndrome antifosfolípido en un periodo de 10 años, las complicaciones obstétricas más frecuentes en las mujeres fueron las pérdidas gestacionales tempranas, la restricción del crecimiento intrauterino y los partos prematuros ^3^.

Actualmente, se sabe que los anticuerpos antifosfolípidos actúan mediante la formación de un complejo antígeno-anticuerpo en la superficie de diferentes células, el cual podría inducir la activación de diferentes vías de señalización, factores de transcripción, expresión de moléculas de adhesión y expresión de proteínas procoagulantes. Estos eventos en conjunto llevan a un estado trombogénico y proinflamatorio, como se ha demostrado en los estudios in vitro, utilizando células endoteliales, monocitos y plaquetas ^4^^-^^6^. También, se ha demostrado que los anticuerpos antifosfolípidos inducen efectos en las células trofoblásticas y, como consecuencia, alteran el desarrollo placentario. En nuestro grupo, se ha estudiado el efecto directo que tienen los anticuerpos antifosfolípidos en mujeres con diferentes manifestaciones clínicas del síndrome antifosfolípido, sobre las células trofoblásticas, las células endoteliales y la interacción trofoblasto-endotelio ^7^^,^^8^.

El óxido nítrico es una molécula conocida por su potente actividad antitrombótica y antiaterogénica; su disminución y el estrés oxidativo están relacionados con la patogénesis del síndrome antifosfolípido ^9^. Se sabe que los anticuerpos antifosfolípidos promueven una perturbación oxidativa y una disfunción mitocondrial, las cuales inducen la activación de una cascada de señalización inflamatoria ^10^. Lo anterior se debe a la capacidad que tienen estos anticuerpos de mediar en la producción de especies reactivas del oxígeno, lo cual podría explicar estos mecanismos patológicos involucrados en el desarrollo del síndrome. Estas moléculas actúan como potentes activadores de vías de señalización incluidas en los eventos proinflamatorios que inducen la expresión de la molécula de adhesión intercelular 1 (ICAM-1), la molécula de adhesión celular vascular (VCAM) y la producción de citocinas proinflamatorias como la interleucina (IL) 6 y la IL-1ß ^11^^-^^13^.

Estos eventos, sumados a la producción de quimiocinas y de mediadores de disfunción endotelial que inducen los anticuerpos antifosfolípidos, dan cuenta de la disfunción endotelial observada en estos pacientes ^10^. Las especies reactivas del oxígeno incluyen formas parcialmente reducidas de oxígeno molecular, tales como el radical hidroxilo (OH), el anión superóxido (O _2_ - ), el peróxido de hidrógeno (H _2_ O _2_ ), los peróxidos lipídicos y el ácido hipocloroso (HCIO).

En condiciones fisiológicas, las células se protegen del daño producido por las especies reactivas del oxígeno mediante mecanismos antioxidantes. El desequilibrio entre los oxidantes y los antioxidantes, lleva al estrés oxidativo que contribuye a la disfunción endotelial ^14^^,^^15^. Además, el estrés oxidativo se ha asociado con la inducción de inmunogenicidad en los antígenos fosfolipídicos característicos del síndrome antifosfolípido ^16^^-^^18^.

La importancia de entender los mecanismos fisiopatológicos involucrados en el desarrollo del síndrome, radica en que su compresión permite optimizar el abordaje terapéutico que debe implementarse en este tipo de pacientes. Por lo tanto, el estudiar el rol del estrés oxidativo como uno de los mecanismos de daño en las células endoteliales, permite aportar al conocimiento actual de la patogénesis del síndrome antifosfolípido.

En consecuencia, el objetivo de este estudio fue evaluar los marcadores de estrés oxidativo en las células endoteliales inducidos por el suero de mujeres con diferentes manifestaciones clínicas del síndrome antifosfolípido y analizar la capacidad antioxidante en los sueros de estas mujeres.

## Materiales y métodos

### Población de estudio

Para incluir en este estudio a las pacientes con síndrome antifosfolípido, se tuvieron en cuenta los criterios internacionales de clasificación revisados de Sapporo, que incluyen: títulos moderados o altos de anticuerpos IgG o IgM anticardiolipina o anti-ß2 glucoproteína I (ß_2_ GPI) o pruebas positivas para el anticoagulante lúpico, persistentes en un intervalo de 12 semanas ^1^.

Como criterios clínicos, se incluyeron la trombosis en cualquier territorio vascular, la morbilidad gestacional o ambas. La morbilidad gestacional se define: como una o más muertes fetales después de la semana 10 de gestación, con morfología normal detectada por ultrasonografía o examen directo; uno o más partos prematuros hasta la semana 34 de gestación como consecuencia de eclampsia, preeclampsia grave o insuficiencia placentaria; o tres o más abortos espontáneos consecutivos antes de la semana 10 de gestación, después de descartar otras causas conocidas de pérdida gestacional ^1^.

Las pacientes con síndrome antifosfolípido u otras enfermedades crónicas diferentes a la autoinmunidad, fueron excluidas del estudio.

Se incluyeron 48 mujeres con anticuerpos antifosfolípidos, las cuales fueron clasificadas en los siguientes grupos, según los criterios clínicos: morbilidad gestacional únicamente (n=10), trombosis vascular únicamente (n=4) y morbilidad gestacional más trombosis vascular (n=10). Como grupos control, se incluyeron mujeres sanas y negativas para anticuerpos antifosfolípidos, con embarazos previos sin complicaciones (n=10), con morbilidad gestacional (n=10) o con trombosis vascular (n=4).

Las pacientes se seleccionaron de la consulta médica del programa “Aborto recurrente” del Grupo de Reproducción de la Facultad de Medicina de la Universidad de Antioquia, y de la Clínica de Anticoagulación del Hospital San Vicente Fundación. Las mujeres sanas se escogieron entre el personal cercano al grupo de investigación.

### Consideraciones éticas

Todas las mujeres aceptaron voluntariamente participar en el estudio y firmaron el consentimiento informado aprobado por el Comité de Ética del Instituto de Investigaciones Médicas de la Universidad de Antioquia, siguiendo las normas establecidas en la Declaración de Helsinki del 3 de octubre de 2000, y las normas del Ministerio de la Protección Social de Colombia, Resolución 8430 de 1993, en la cual se exige el respeto de los derechos y la dignidad de los pacientes, sin manipulación ni alteración de la información en el manejo de las historias clínicas.

### Obtención de muestras

Mediante venopunción en el antebrazo, se recolectó sangre periférica de cada una de las mujeres en tubos secos libres de anticoagulante (Fenwal, USA). La muestra se centrifugó a 460g por 15 minutos para separar el suero y se almacenó a -80 °C hasta su uso. Dada la heterogeneidad de los anticuerpos antifosfolípidos, se elaboró un grupo (pool) con los sueros de cada grupo de estudio.

En los sueros de los grupos de estudio, se evaluó la presencia de anticuerpos IgG anticardiolipina con el estuche comercial Aeskulisa Cardiolipin-GM™ (Aesku Diagnostics, Germany), y de anticuerpos IgG anti-ß_2_ glucoproteína I, con el estuche Aeskulisa ß_2_ -Glyco-GM™ (Aesku Diagnostics). En el plasma de las mismas mujeres, se determinó la positividad del anticoagulante lúpico por el tiempo parcial de tromboplastina activado, usando el estuche comercial APTT-SP™ (Instrumentation Laboratory Bedford, USA), y también, el tiempo de víbora de Russell, con el estuche comercial LAC Screen™ para la tamización y el LAC Confirm™ para la prueba confirmatoria (Rochem Biocare, Colombia).

También, se evaluó la positividad de los anticuerpos IgG anticardiolipina y de otros anticuerpos antifosfolípidos ‘no criterio’, utilizando un ELISA estandarizado en el laboratorio a partir de la técnica publicada por Kwak, *et al*. ^19^.

En microplatos de poliestireno de 96 pozos con fondo en U Maxisorp Nunc™ (Thermo Fisher Scientific, USA), se cubrieron con 30 µl de una suspensión de 50 µg/ml de cardiolipina en etanol (Sigma-Aldrich, USA) o 50 µg/ml en metanol de los otros cinco fosfolípidos (fosfatidilglicerol, ácido fosfatídico, fosfatidilserina, anti-fosfatidiletanolamina y fosfatidilinositol) (Sigma-Aldrich).

Los platos se dejaron secar a 4 °C durante 18 horas, luego se lavaron con solución tampón PBS (Phosphate Buffered Saline) 1X y se bloquearon con una solución de PBS y suero de bovino adulto (SBA) al 20 % (Gibco, USA) durante 90 minutos a temperatura ambiente, protegidos de la luz.

Después de otro lavado con solución tampón PBS, se agregaron por duplicado 50 µl de los sueros de las pacientes a una dilución de 1:50 en suero de bovino adulto al 20 %, se incubaron dos horas a temperatura ambiente en la oscuridad y, después, se lavaron tres veces con solución PBS.

Se agregaron 50 µl del segundo anticuerpo anti-IgG humana conjugado con fosfatasa alcalina (Invitrogen, USA) a una dilución 1:1.000 en suero de bovino adulto al 20 %, se incubaron por 90 minutos y se lavaron como se indicó anteriormente.

Se agregaron 50 µl de la solución reveladora de paranitrofenilfosfato (Sigma-Aldrich) a 1 mg/ml en solución sustrato (dietanolamina al 10 %, MgCl_2_ al 0,005 % y azida de sodio al 0,02 %, pH 9,8). La reacción se paró con 50 µl de una solución 3M de NaOH.

La densidad óptica de cada pozo se determinó usando un lector de microplatos para ELISA (Multiskan FC™, Thermo Scientific, USA) a una longitud de onda de 405 a 410 nm. En cada plato se incluyó un blanco con solución de revelado y solución de parada, un control positivo y un control negativo para cada uno de los antígenos. Además, se incluyó un control de unión inespecífica de los sueros, colocando cada uno de los sueros en un pozo sin antígeno, cuyo valor se resta del promedio de las densidades ópticas de las muestras.

Se consideraron positivos los valores de densidad óptica iguales o mayores del 25 % de la densidad óptica del control positivo.

Para clasificar a las mujeres como positivas para cualquiera de los anticuerpos antifosfolípidos, ellas debían ser positivas en dos ocasiones, con un intervalo mínimo de 12 semanas.

### Cultivo de células endoteliales

Las células endoteliales de vena de cordón umbilical humano se aislaron a partir de cordones umbilicales de mujeres con un embarazo normal, como se describió previamente ^8^.

Brevemente, se perfundió la vena umbilical con una aguja pericraneal para adicionar colagenasa de tipo I (Invitrogen) y se incubó a 37 °C por 20 minutos. El contenido de la vena umbilical se centrifugó a 300g por cinco minutos y a 22 °C. El botón de células se cultivó en frascos de 75 cm^2^ con filtros Nunc™ (Thermo Fisher Scientific), con medio de cultivo basal de células endoteliales con suplemento (Promocell, Germany) al 2% de suero bovino fetal (Gibco). Finalmente, se removieron los detritos para optimizar el cultivo.

Las células endoteliales se utilizaron en los pases 1 y 2. En platos de 24 pozos, se adicionaron 5 x 105 células endoteliales de vena de cordón umbilical humano por pozo y se estimularon por 24 horas con 10 % de suero de los grupos de mujeres del estudio, para determinar su efecto sobre la producción de especies reactivas del oxígeno intracelulares, anión superóxido mitocondrial y lipoperoxidación de membrana por citometría de flujo.

Las células se desprendieron con 0,025 % de tripsina (Sigma-Aldrich), la cual fue inactivada con Opti-MEM™ (Gibco) en suero de bovino adulto al 10 %. Todas las lecturas se hicieron en el citómetro de flujo LSR Fortessa™ (Becton Dickinson, USA). Se obtuvieron 10.000 eventos en cada una de las lecturas realizadas.

### Determinación de la pureza de las células endoteliales

A los cuatro días de cultivo, se evaluó la pureza de las células endoteliales mediante la detección de la expresión de la molécula de adhesión CD31 o PECAM-1 (molécula de adhesión celular endotelial plaquetaria 1). La molécula CD31 es útil para evaluar la pureza de las células endoteliales, debido a que es una glucoproteína característica de estas células, que proporciona estabilidad a la monocapa y adhesión intercelular, por lo cual se ha empleado como herramienta para detectar o aislar células endoteliales ^12^^,^^13^. 

La expresión de la molécula CD31 se evaluó por citometría de flujo y por microscopía de fluorescencia. Para ambas técnicas, las células se marcaron con la CD31 conjugada con fluoresceína FITC™ (Thermo Fisher Scientific), adicionada a las células en una dilución de 1:100 de solución tampón PBS e incubación durante 40 minutos, y los núcleos se tiñeron con yoduro de propidio (Thermo Fisher Scientific).

La citometría se realizó como se describió anteriormente en el citómetro de flujo LSR Fortessa™ (Becton Dickinson); para la microscopía de fluorescencia, las células se permeabilizaron utilizando Cytofix™ y la solución tampón Perm/Wash™ (Biosciences BD, USA), y la autofluorescencia fue bloqueada utilizando 50 mM de cloruro de amonio.

Finalmente, la monocapa de células se visualizó en un microscopio de fluorescencia invertido Zeiss Axio Vert.A1™ (Carl Zeiss Microscopy, Germany) con el filtro GFP, con una excitación BP 475/40 y una emisión BP 530/50, y con el filtro RFP, con una excitación BP de 572/25 y una emisión BP 629/62. El microscopio estaba adaptado con una cámara DS-fi1™ (Nikon Corporation, Japón) y se utilizó un aumento de 20X.

### Detección de especies reactivas del oxígeno

La detección de especies reactivas del oxígeno intracelulares, como el radical hidroxilo y el peróxido de hidrógeno, se hizo con el compuesto diacetato de diclorofluoresceína (Sigma-Aldrich). Este compuesto es desacetilado por las esterasas celulares a diclorofluoresceína (DCFH) que no es fluorescente, pero, cuando interactúa con las especies reactivas del oxígeno, se transforma en el compuesto fluorescente DCF.

Las células se lavaron por centrifugación a 580g por cinco minutos con 600 µl de solución tampón PBS (Amresco, USA) y se les adicionaron 0,05 µM de DCFH-DA y 0,5 µM de yoduro de propidio, (Thermo Fisher Scientific). Como control positivo para detectar especies reactivas del oxígeno intracelulares, se estimularon las células endoteliales de vena de cordón umbilical humano con peróxido de hidrógeno (Sigma-Aldrich) a concentraciones de 0,5 y 1 mM. La lectura se hizo en el citómetro, como se indicó anteriormente.

### Producción del anión superóxido

El anión superóxido se acumula en el interior de la mitocondria como resultado del metabolismo celular de la cadena transportadora de electrones y presenta un fuerte potencial negativo que permite su interacción con el compuesto lipofílico de carga positiva trifenilfosfonio, disponible de manera comercial como MitoSOX™ (Invitrogen) ^20^. La sonda MitoSOX se adicionó a las células endoteliales en una concentración de 0,02 µM. Para evaluar la viabilidad de las células productoras de anión superóxido, se usó la sonda eFluor™ (Thermo Fisher Scientific) en una concentración de 0,1 µM. La lectura se hizo en el citómetro.

### Evaluación de lipoperoxidación

El BODIPY C11™ (Life Technology, USA) es una molécula lipofílica que posee un grupo 4-fenil-1,3-butadienilo unido a un pirrol; el butadienilo puede someterse a oxidación por lípidos oxidados, lo que resulta en un desplazamiento del pico de emisión de fluorescencia de ~590 nm a ~510 nm ^21^^,^^22^. El BODIPY C11™ se adicionó a las células en una concentración de 0,825 µM en 1.200 µl de solución tampón PBS (Amresco). La lectura se hizo en el citómetro.

### Cuantificación de actividad antioxidante en los sueros de las pacientes

La enzima paraoxonasa 1 es una arilesterasa, organofosfatasa, que se encuentra localizada en la superficie de las lipoproteínas de alta densidad (HDL) y tiene la capacidad de disminuir la oxidación de las lipoproteínas de baja densidad (LDL); además, participa en la prevención y el equilibrio del estrés oxidativo ^23^.

La actividad de esta enzima se evaluó usando el estuche comercial EnzCheck™ de estudio fluorométrico de paraoxonasa (Invitrogen). El estudio presenta un límite de detección, aproximadamente, de 50 mU/ml y es 10 veces más sensible que los métodos colorimétricos clásicos ^24^.

La intensidad de la luz emitida se determinó usando el Varioskan Flash Multimode Reader™ (Thermo Fisher Scientific) en los filtros de 360 y 450 nm para excitación y emisión, respectivamente, cada 15 minutos por una hora a 37 °C.

La actividad enzimática se calculó restando la fluorescencia de los controles negativos a las muestras y se utilizó la ecuación de la curva estándar para determinar la cantidad de producto fluorescente de cada muestra. Finalmente, la cantidad del producto fluorescente formado se transformó en unidades de paraoxonasa, donde cada unidad de paraoxonasa genera un nmol de producto fluorescente por minuto a 37 °C.

La capacidad antioxidante total también se evaluó con el reactivo 2,2-difenil1-picrilhidrazilo (DPPH) (Sigma-Aldrich) que dona un hidrógeno a los antioxidantes presentes ^25^. El reactivo DPPH se mezcló con metanol para obtener una solución 0,1 mM y se almacenó en la oscuridad a -80 ºC. A los sueros se les eliminaron las proteínas, como se ha descrito previamente ^26^, y se almacenaron a -80 ºC, hasta su uso. A las muestras se les adicionó el DPPH y se colocaron en un mezclador de vórtice (vortex mixer); posteriormente, se incubaron en la oscuridad durante una hora a temperatura ambiente.

La absorbancia se detectó a 515 nm por espectrofotometría en el lector de barrido espectral Varioskan Flash™. La capacidad antioxidante total fue proporcional a la disminución de la absorbancia del reactivo DPPH por las muestras.

### Análisis estadístico

Los resultados de la citometría se analizaron en el programa FlowJo™ (TreeStar, Inc. USA); los valores se indican como intensidad mediana de fluorescencia y fueron normalizados con respecto al control de mujeres con embarazo previo sin complicaciones. A los datos obtenidos se les hizo la prueba de normalidad de Shapiro-Wilk y, según su distribución, se determinó la significancia estadística (p<0,05) con una un análisis de varianza (ANOVA) de una vía y un ‘post-test’ de Holm-Sidak, o con una prueba de KruskalWallis y un ‘post-test’de Dunns.

Los análisis estadísticos se hicieron en el programa GrandPad Prism 6™ (Graph Pad Software, Inc. USA).

## Resultados

El grupo de mujeres con morbilidad gestacional, trombosis venosa y anticuerpos antifosfolípidos tuvieron positivas todas las pruebas de laboratorio incluidas en los criterios de Sapporo ^1^. En el grupo de mujeres con morbilidad gestacional y anticuerpos antifosfolípidos se detectaron anticuerpos anticardiolipina persistentes mediante ELISA estandarizada en el laboratorio, pero no por los estuches comerciales. A pesar de esto, se decidió incluir a estas pacientes en el estudio, ya que también eran positivas para otros anticuerpos ‘no criterio’ dirigidos a fosfolípidos cargados negativamente y evaluados también por ELISA estandarizada en el laboratorio. La inclusión de estos anticuerpos como criterios de clasificación, aún se encuentra en discusión ^27^.

En trabajos previos de nuestro grupo de investigación, hemos encontrado efecto de los sueros de este grupo de pacientes sobre diferentes funciones de las células trofoblásticas ^7^^,^^8^^,^^28^. Las mujeres del grupo con morbilidad gestacional sin anticuerpos antifosfolípidos y las mujeres sanas con embarazo previo sin complicaciones del grupo control, fueron negativas para todas las pruebas de laboratorio de anticuerpos antifosfolípidos (cuadro 1).

**Cuadro 1:** Características de las mujeres incluidas en el estudio, Hospital Universitario Dr. Luis Razetti, Barcelona, estado Anzoátegui, Venezuela

Parámetro		SHN	MG/aAFL-	TV/aAFL-	MG/aAFL+	TV/aAFL+	MG/TV/aFL+
		(n=10)	(n=10)	(n=4)	(n=10)	(n=4)	(n=10)
	
							
Edad (media de años ± DE)		35,5 ± 1,7	30,9 ± 1,9	35,0 ± 7,4	34,8 ± 2,4	29,7 ± 7,2	37,2 ± 1,8
Pérdidas gestacionales (media y rango)							
≤10 semanas de gestación		0	1,9 (1-3)	0	1,2 (1-2)	0	1,3 (1-5)
>10 semanas de gestación		0	0,7 (1-3)	0	0,9 (1-4)	0	1,7 (1-5)
Preeclampsia <34 semanas (número de pacientes)		0	0	0	0	0	6
Restricción del crecimiento intrauterino (número de							
pacientes)		0	0	0	1	0	1
Trombosis arterial/venosa (número de pacientes)		0	0	4	0	4	10
Anticoagulante lúpico (promedio ± DE)							
Valor positivo >1.2		1,06 ± 0,05	1,13 ± 0,01	1,12 ± 0,04	1,17 ± 0,13	2,32 ± 0,31	2,38 ± 0,28
Anti-β2 glucoproteína I (U/ml)							
Valor positivo >15 U/ml		10,42	4,21	5,04	6,12	63,4	100,7
Anticardiolipina por el ELISA comercial Aesku							
Valor positivo>15 GPL/ml		5,63	4,91	8,53	8,30	156,7	300,2
Anticardiolipina por ELISA estandarizado en el laboratorio									
(% del control)							
Valor positivo >25 %		24,0	19	9,6	100	60,2	49,1
Anticuerpos antifosfolípidos ‘no criterio’” (% del control)							
Valor positivo >25 %	GPG	17,0	24,0	10,0	176,9	63,6	66,5
	GPA	0,9	3,7	0	23,0	60,5	114,8
	GPS	0,9	4,6	0,8	20,5	28,9	18,2
	GPE	4,3	16,8	0	25,5	21,1	3,6
	GPI	4,9	8,9	1	18,7	17,4	15,1

SHN: mujeres negativas para anticuerpos antifosfolípidos (aAFL) y con embarazos previos sin complicaciones; MG/aAFL-: mujeres con morbilidad gestacional, negativas para anticuerpos antifosfolípidos; TV/ aAFL-: mujeres con trombosis vascular, negativas para anticuerpos antifosfolípidos; MG/aAFL+: mujeres con morbilidad gestacional sola y anticuerpos antifosfolípidos no-criterio positivos. TV/ aAFL+: mujeres con trombosis vascular sola y anticuerpos antifosfolípidos positivos; MG/TV/ aAFL+: mujeres con trombosis vascular y morbilidad gestacional, positivas para anticuerpos antifosfolípidos. DE: desviación estándar; GPL: unidades estándar de inmunoglobulina G anticardiolipina, GPG: inmunoglobulina G anti-fosfatidilglicerol, GPA: inmunoglobulina G anti-ácido fosfatídico, GPS: inmunoglobulina G antifosfatidilserina, GPE: inmunoglobulina G anti-fosfatidiletanolamina, GPI: inmunoglobulina G anti-fosfatidilinositol

### Cultivo de células endoteliales de vena de cordón umbilical humano

En este estudio, las células endoteliales de vena de cordón umbilical humano se obtuvieron a partir de cordones umbilicales provenientes del parto de mujeres con un embarazo normal, como se describió previamente. Después del aislamiento y, aproximadamente, de cuatro días de cultivo, se evaluó la pureza de las células endoteliales mediante la detección de la expresión de la molécula de adhesión CD31.

**Figura 1.**
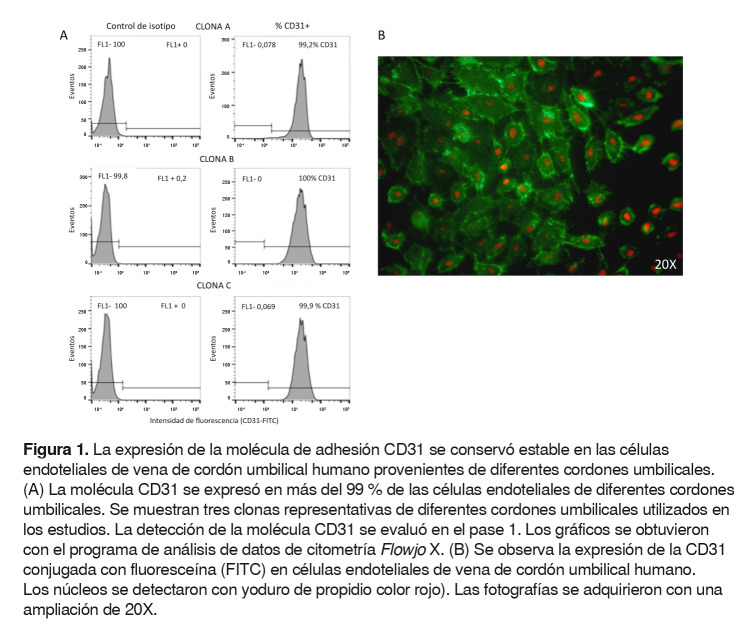
La expresión de la molécula de adhesión CD31 se conservó estable en las células endoteliales de vena de cordón umbilical humano provenientes de diferentes cordones umbilicales. (A) La molécula CD31 se expresó en más del 99 % de las células endoteliales de diferentes cordones umbilicales. Se muestran tres clonas representativas de diferentes cordones umbilicales utilizados en los estudios. La detección de la molécula CD31 se evaluó en el pase 1. Los gráficos se obtuvieron con el programa de análisis de datos de citometría Flowjo X. (B) Se observa la expresión de la CD31 conjugada con fluoresceína (FITC) en células endoteliales de vena de cordón umbilical humano. Los núcleos se detectaron con yoduro de propidio color rojo). Las fotografías se adquirieron con una ampliación de 20X.

En las imágenes representativas de tres cordones umbilicales, se puede observar que la expresión de la CD31 en las células provenientes de diferentes mujeres, se mantuvo estable en más del 99 % de las células (figura 1A). Por microscopía de fluorescencia, también se encontró positividad de la CD31 en los cultivos de células endoteliales de vena de cordón umbilical humano purificados para los estudios posteriores (figura 1B).

### Efecto de los sueros de los grupos de estudio sobre la viabilidad celular

**Figura 2.**
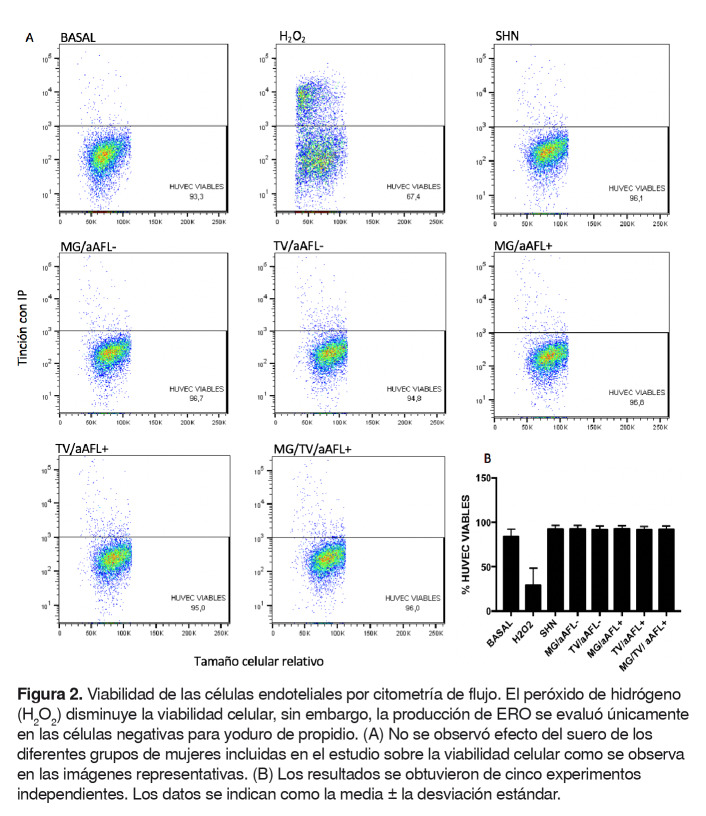
Viabilidad de las células endoteliales por citometría de flujo. El peróxido de hidrógeno (H_2_ O_2_ ) disminuye la viabilidad celular, sin embargo, la producción de ERO se evaluó únicamente en las células negativas para yoduro de propidio. (A) No se observó efecto del suero de los diferentes grupos de mujeres incluidas en el estudio sobre la viabilidad celular como se observa en las imágenes representativas. (B) Los resultados se obtuvieron de cinco experimentos independientes. Los datos se indican como la media ± la desviación estándar.

En todos los estudios de estrés oxidativo, se incluyó yoduro de propidio o e-Fluor™ para excluir las células muertas de los análisis de detección de producción de especies reactivas del oxígeno intracelulares, anión superóxido y peroxidación lipídica. Se observó que los sueros de los grupos de mujeres del estudio no afectaron la viabilidad celular (figura 2).

### Producción de especies reactivas del oxígeno intracelulares

**Figura 3.**
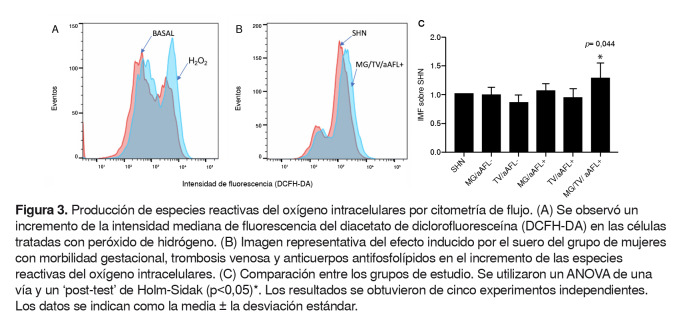
Producción de especies reactivas del oxígeno intracelulares por citometría de flujo. (A) Se observó un incremento de la intensidad mediana de fluorescencia del diacetato de diclorofluoresceína (DCFH-DA) en las células tratadas con peróxido de hidrógeno. (B) Imagen representativa del efecto inducido por el suero del grupo de mujeres con morbilidad gestacional, trombosis venosa y anticuerpos antifosfolípidos en el incremento de las especies reactivas del oxígeno intracelulares. (C) Comparación entre los grupos de estudio. Se utilizaron un ANOVA de una vía y un ‘post-test’ de Holm-Sidak (p<0,05) *. Los resultados se obtuvieron de cinco experimentos independientes. Los datos se indican como la media ± la desviación estándar.

En las células estimuladas con el suero de los diferentes grupos de pacientes, se encontró que, específicamente, el suero del grupo de mujeres con morbilidad gestacional, trombosis venosa y anticuerpos antifosfolípidos provoca un aumento significativo (p=0,044) en la concentración de especies reactivas del oxígeno intracelulares en comparación con el suero del grupo control (figuras 3A y B).

El control implementado de peróxido de hidrógeno incrementó la intensidad mediana de fluorescencia del compuesto DCFH-DA en las células endoteliales, en contraste con las células cultivadas con Opti-MEM™ (Gibco), lo que indica que este sistema logra detectar el aumento de especies reactivas del oxígeno intracelulares en contraste con la producción basal (figuras 3B y C).

### Detección del anión superóxido y peroxidación lipídica

**Figura 4.**
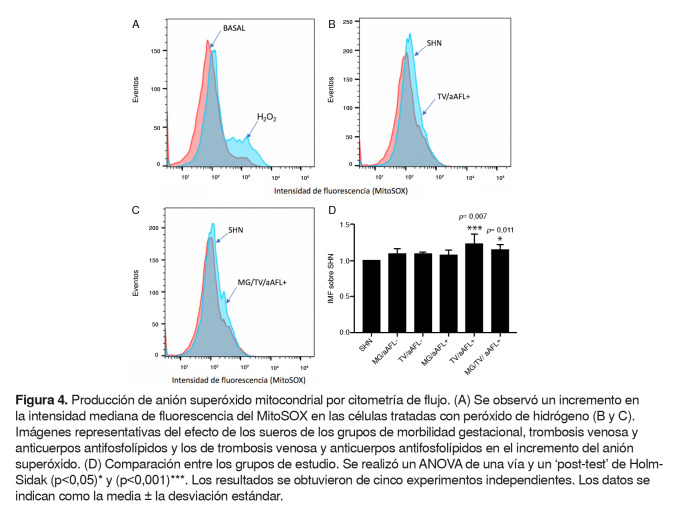
Producción de anión superóxido mitocondrial por citometría de flujo. (A) Se observó un incremento en la intensidad mediana de fluorescencia del MitoSOX en las células tratadas con peróxido de hidrógeno (B y C). Imágenes representativas del efecto de los sueros de los grupos de morbilidad gestacional, trombosis venosa y anticuerpos antifosfolípidos y los de trombosis venosa y anticuerpos antifosfolípidos en el incremento del anión superóxido. (D) Comparación entre los grupos de estudio. Se realizó un ANOVA de una vía y un ‘post-test’ de HolmSidak (p<0,05) y (p<0,001) ***. Los resultados se obtuvieron de cinco experimentos independientes. Los datos se indican como la media ± la desviación estándar.

El control positivo de peróxido de hidrógeno provocó un aumento de la intensidad mediana de fluorescencia del MitoSOX, en contraste con la producción basal de anión superóxido (figura 4A)

El suero de mujeres con morbilidad gestacional, trombosis venosa y anticuerpos antifosfolípidos y el de mujeres con trombosis venosa y anticuerpos antifosfolípido indujo un incremento significativo en la intensidad mediana de fluorescencia del MitoSOX, en comparación con el control de mujeres con embarazo previo sin complicaciones (p=0,011 y p=0,0007, respectivamente) (figuras 4 B, C y D). 

**Figura 5.**
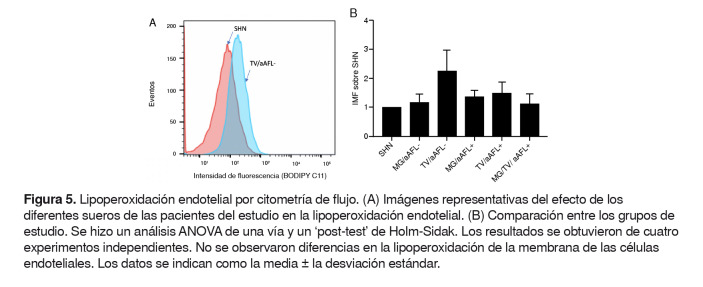
Lipoperoxidación endotelial por citometría de flujo. (A) Imágenes representativas del efecto de los diferentes sueros de las pacientes del estudio en la lipoperoxidación endotelial. (B) Comparación entre los grupos de estudio. Se hizo un análisis ANOVA de una vía y un ‘post-test’ de Holm-Sidak. Los resultados se obtuvieron de cuatro experimentos independientes. No se observaron diferencias en la lipoperoxidación de la membrana de las células endoteliales. Los datos se indican como la media ± la desviación estándar.

No se observó diferencia en la inducción de lipoperoxidación de las membranas de las células endoteliales, entre los diferentes grupos de estudio (figuras 5 A y B).

### Capacidad antioxidante en los sueros de los pacientes

**Figura 6.**
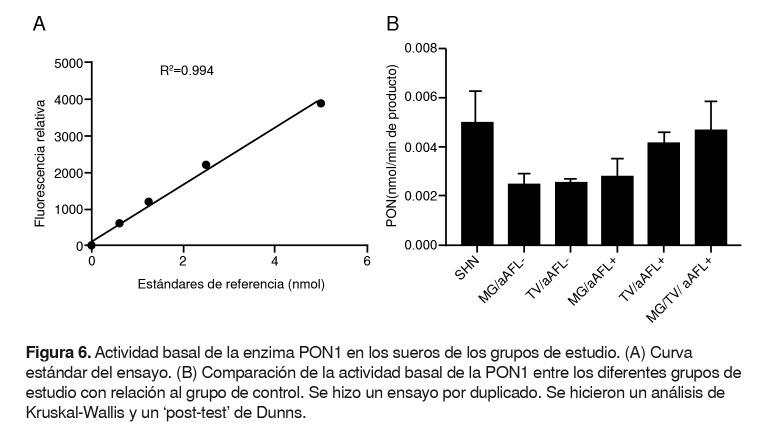
Actividad basal de la enzima PON1 en los sueros de los grupos de estudio. (A) Curva estándar del ensayo. (B) Comparación de la actividad basal de la PON1 entre los diferentes grupos de estudio con relación al grupo de control. Se hizo un ensayo por duplicado. Se hicieron un análisis de Kruskal-Wallis y un ‘post-test’ de Dunns.

No se encontró diferencia en la actividad basal de la enzima PON-1 ni en el porcentaje de la capacidad antioxidante total de los sueros de los diferentes grupos de estudio, comparados con el grupo de control (figuras 6 y 7).

**Figura 7.**
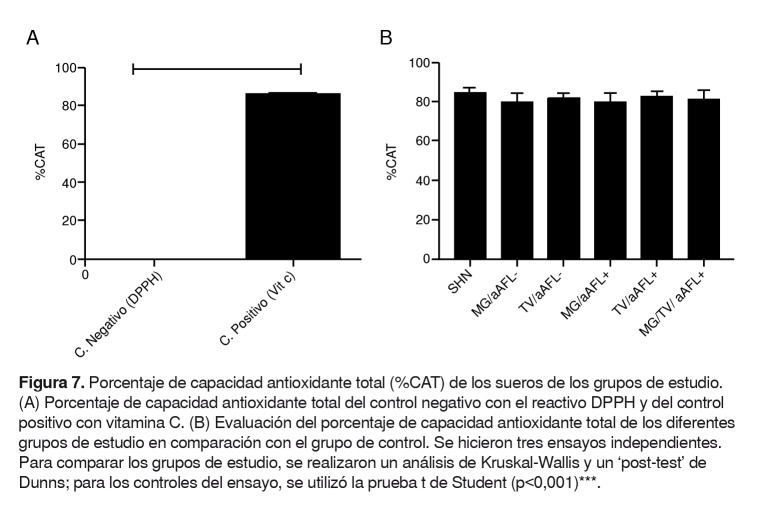
Porcentaje de capacidad antioxidante total (%CAT) de los sueros de los grupos de estudio. (A) Porcentaje de capacidad antioxidante total del control negativo con el reactivo DPPH y del control positivo con vitamina C. (B) Evaluación del porcentaje de capacidad antioxidante total de los diferentes grupos de estudio en comparación con el grupo de control. Se hicieron tres ensayos independientes. Para comparar los grupos de estudio, se realizaron un análisis de Kruskal-Wallis y un ‘post-test’ de Dunns; para los controles del ensayo, se utilizó la prueba t de Student (p<0,001)***.

## Discusión

Se ha descrito que en la inmunopatogénesis del síndrome antifosfolípido obstétrico, el daño placentario puede producirse por la activación del sistema del complemento inducida por los anticuerpos antifosfolípidos en los neutrófilos, los cuales, en consecuencia, producen especies reactivas del oxígeno que actúan sobre las células endoteliales, causando daño y activación lo que favorece la pérdida fetal ^29^^,^^30^.

En el síndrome antifosfolípido, la producción de especies reactivas del oxígeno también provoca disfunción endotelial que es determinante para desencadenar los eventos trombóticos al provocar un estado proadhesivo y activar las vías de señalización que involucran a la proteína cinasa activada por mitógenos (MAKP) p38 que, a su vez, controla la expresión de la molécula de adhesión ICAM-1, lo cual favorece el estado inflamatorio por la adhesión de monocitos al endotelio ^12^.

En este trabajo se evaluó el efecto directo del suero de mujeres con diferentes manifestaciones clínicas del síndrome antifosfolípido sobre las células endoteliales, lo cual contribuye al entendimiento del mecanismo patológico de los anticuerpos antifosfolípidos asociado a las características de las pacientes. Por el contrario, en trabajos previos de otros autores, se ha evidenciado un incremento de las especies reactivas del oxígeno inducida por los anticuerpos antifosfolípidos, pero no es clara su asociación con las manifestaciones clínicas del síndrome antifosfolípido ^12^^).^

En el presente trabajo, se encontró que el suero de ambos grupos de pacientes con trombosis, con morbilidad gestacional o sin ella (trombosis venosa y anticuerpos antifosfolípidos y morbilidad gestacional, trombosis venosa y anticuerpos antifosfolípidos), induce un incremento del anión superóxido mitocondrial. Sin embargo, el suero del grupo de mujeres con morbilidad gestacional, trombosis venosa y anticuerpos antifosfolípidos también provoca la producción intracelular de especies reactivas del oxígeno.

En la gestación, se requiere la producción de especies reactivas del oxígeno para el desarrollo placentario, porque puede regular la activación de factores de transcripción involucrados en la proliferación, la invasión trofoblástica y la angiogénesis ^31^. Las especies reactivas del oxígeno también regulan la autofagia y la apoptosis, necesarias para la homeostasis placentaria ^31^. Sin embargo, en contextos patológicos, un desequilibrio en la producción de dichas especies puede ser la causa de una placentación defectuosa que genera morbilidad gestacional. En reportes recientes, se ha planteado que, en los diferentes grupos de pacientes con síndrome antifosfolípido y con anticuerpos antifosfolípidos positivos, se producen manifestaciones clínicas de trombosis, morbilidad gestacional o ambas, según la concentración de dichos anticuerpos ^32^.

En el presente trabajo, específicamente en el grupo de mujeres con morbilidad gestacional, trombosis venosa y anticuerpos antifosfolípidos, se presentaron mayores niveles de anti-ß2 GPI y de anticardiolipina (cuadro 1), y mayor inducción de estrés oxidativo, lo que indica que los altos títulos de anticuerpos antifosfolípidos y la presencia de ambas manifestaciones clínicas desencadenan importantes efectos deletéreos sobre el endotelio.

Se ha demostrado que la generación del anión superóxido incrementa la activación de los receptores de tipo toll 7 y 8 (TLR-7 y 8) que, a su vez, aumentan la producción de citocinas inflamatorias ^33^. Además, en los monocitos deficientes de NADPH oxidasa 2, estos mismos autores evidenciaron una enzima que produce superóxido y que no se incrementaba la producción del factor tisular como reacción a los anticuerpos antifosfolípidos ^33^^,^^34^. La producción del factor tisular se incrementa en los pacientes con síndrome antifosfolípido en un estado de hipercoagulabilidad ^35^. Sin embargo, no es claro si estas vías intracelulares varían con las presentaciones clínicas del síndrome antifosfolípido que, como se observó en este trabajo, pueden provocar estrés oxidativo por diferentes mecanismos.

En los modelos en ratón, se encontró que la inyección de anticuerpos antifosfolípidos anti-ß_2_ GPI induce estrés oxidativo, el cual se detecta en el ventrículo izquierdo por disminución de la relación entre el glutatión reducido y el oxidado, asociada con el incremento de la molécula de adhesión celular vascular (VCAM) y de la selectina E soluble (CD62E), y disminución de la relajación dependiente del endotelio y de la expresión de la sintasa endotelial de óxido nítrico (eNOS) en las arterias mesentéricas ^36^.

Lo anterior sugiere que el incremento del estrés oxidativo inducido por los anticuerpos antifosfolípidos de los pacientes con síndrome antifosfolípido, altera la funcionalidad de la sintasa endotelial de óxido nítrico; sin embargo, no es claro si estos eventos se asocian con trombosis ^36^. No obstante, se sabe que, en el endotelio tratado con anticuerpos antifosfolípidos de pacientes con trombosis, se reduce la fosforilación de dicha sintasa endotelial, dependiendo del receptor 2 de la apopolipoproteína E ^37^. En estudios posteriores, podría considerarse la evaluación de una posible relación del estrés oxidativo y la disminución de la activación de la sintasa endotelial de óxido nítrico, ya que ambos eventos son inducidos por anticuerpos antifosfolípidos de pacientes con síndrome antifosfolípido que tienen trombosis, como se encontró en este trabajo y en otros reportes ^37^.

Por otra parte, no se evidenciaron diferencias en la actividad de la enzima PON1 en los sueros de los grupos de mujeres incluidas que, según otros reportes, se disminuye en pacientes con síndrome antifosfolípido primario y en modelos en ratón inyectados con anticuerpos antifosfolípidos ^38^^,^^39^. Tampoco se observaron alteraciones en la capacidad antioxidante total, lo que indica que el estrés oxidativo endotelial en este síndrome no estaría asociado con un desequilibrio entre oxidantes y los mecanismos antioxidantes evaluados en este trabajo, como se ha planteado en otros contextos ^40^.

Los resultados encontrados permiten concluir que el estrés oxidativo mitocondrial en el endotelio se asocia con la presencia de trombosis; sin embargo, cuando esta se presenta con morbilidad gestacional, también se genera estrés oxidativo intracelular, lo que sugiere que, en las pacientes con síndrome antifosfolípido que presentan trombosis y morbilidad gestacional, los anticuerpos antifosfolípidos podrían tener efectos más nocivos.

Aún no se ha esclarecido el efecto que puedan tener las especies reactivas del oxígeno inducidas por los anticuerpos antifosfolípidos de los pacientes con síndrome antifosfolípido que presentan trombosis, en la activación de vías intracelulares en el endotelio.
